# Decoding emotional responses to AI-generated architectural imagery

**DOI:** 10.3389/fpsyg.2024.1348083

**Published:** 2024-03-12

**Authors:** Zhihui Zhang, Josep M. Fort, Lluis Giménez Mateu

**Affiliations:** Escola Tècnica Superior d'Arquitectura de Barcelona, Universitat Politècnica de Catalunya, Barcelona, Spain

**Keywords:** artificial intelligence, emotional perception, architectural imagery, emotional rendering, architectural design, affective computing

## Abstract

**Introduction:**

The integration of AI in architectural design represents a significant shift toward creating emotionally resonant spaces. This research investigates AI's ability to evoke specific emotional responses through architectural imagery and examines the impact of professional training on emotional interpretation.

**Methods:**

We utilized Midjourney AI software to generate images based on direct and metaphorical prompts across two architectural settings: home interiors and museum exteriors. A survey was designed to capture participants' emotional responses to these images, employing a scale that rated their immediate emotional reaction. The study involved 789 university students, categorized into architecture majors (Group A) and non-architecture majors (Group B), to explore differences in emotional perception attributable to educational background.

**Results:**

Findings revealed that AI is particularly effective in depicting joy, especially in interior settings. However, it struggles to accurately convey negative emotions, indicating a gap in AI's emotional range. Architecture students exhibited a greater sensitivity to emotional nuances in the images compared to non-architecture students, suggesting that architectural training enhances emotional discernment. Notably, the study observed minimal differences in the perception of emotions between direct and metaphorical prompts among architecture students, indicating a consistent emotional interpretation across prompt types.

**Conclusion:**

AI holds significant promise in creating spaces that resonate on an emotional level, particularly in conveying positive emotions like joy. The study contributes to the understanding of AI's role in architectural design, emphasizing the importance of emotional intelligence in creating spaces that reflect human experiences. Future research should focus on expanding AI's emotional range and further exploring the impact of architectural training on emotional perception.

## 1 Introduction

The integration of artificial intelligence (AI) in architectural design marks a significant shift in our engagement with the built environment. This integration challenges traditional perceptions of architecture as a fusion of human emotion and spatial design, a concept echoed by Corbusier and Etchells (2014). The impact of architectural elements such as color, light, and space on human emotions and behaviors, recognized in previous studies (Mehrabian and Russell, 1974; Pallasmaa, 2012; Zhang et al., 2022), underscores the significance of this evolution.

The rise of AI-generated architectural imagery sparks debates within architectural and psychological circles about AI's capacity to evoke emotional resonance akin to human-designed structures (Botros et al., 2023). Public opinion is divided: while some critics argue AI lacks the inherent human touch necessary for genuine emotional engagement (Daniele and Song, 2019; Cetinic and She, 2022; Demmer et al., 2023), others advocate for AI's potential to elicit complex emotional responses (Bagozzi et al., 2022; Cheng et al., 2022). This dichotomy opens up broader inquiries into the role of emotion and perception in AI-enhanced art and design.

Our research delves into the psychological aspects of responses to AI-enhanced architectural imagery. Drawing on interdisciplinary research in human-AI interaction (Ashlock et al., 2023; Zhang et al., 2023), we analyze the emotional reactions of individuals with varying architectural expertise to AI-generated images, including both interior and exterior visualizations. We also investigate the effect of different AI image generation methods on emotional perception (Zhao, 2016). Additionally, the research explores the implications of AI use in architectural education, design practices, and technology evolution, raising philosophical and ethical questions about the interplay between artificial creations and natural human responses.

In conclusion, while acknowledging the limitations of current research, we propose future research directions focused on the synergistic relationship between AI and human designers, and the cultural and social nuances of emotional resonance in AI-generated designs. Our study aims to decode the complex emotional responses triggered by AI in architectural design, contributing to a deeper understanding of behavioral sciences at the intersection of technology and creativity (Pressman, 2001).

## 2 Literature review

### 2.1 AI-generated images in architecture

Recent advancements in AI-generated imagery have significantly impacted the intersection of technology and creativity. Göring underscores the capability of AI generators to produce images that are not only highly realistic but also visually appealing, highlighting that the outcome largely depends on the methodology and precision of the text prompts used (Göring et al., 2023). Similarly, Chen delves into the use of deep learning technologies for creating artistic illustrations from concise text descriptions, showcasing AI's ability for style transfer aligned with narrative content, which illustrates the adaptability of AI to various artistic requirements (Chen et al., 2020).

Lu et al. (2024) presents a compelling discovery that humans have a 38.7% success rate in distinguishing real photographs from those generated by AI, suggesting AI's potential to revolutionize visual expression across industries by mimicking reality closely. This could lead to a future where AI not only augments human creativity but also enriches aesthetic environments.

In architecture, Lee et al.'s (2024) research demonstrates AI's capacity to articulate a wide array of design styles in interior spaces, enhancing spatial layouts with specific features, and embodying the design ethos of distinguished architects. Zhang further investigates AI's role as a pivotal tool in architectural design, offering a variety of design solutions and driving innovation. While acknowledging AI's strengths in fostering attractiveness and creativity, Zhang et al. (2023) also notes areas for improvement in authenticity and coherence of the generated designs. Similarly, Akhtar and Ramkumar (2023) views AI more as a collaborator than a substitute in the architectural design process, suggesting that architects can leverage AI to realize innovative solutions and simplify complex tasks.

### 2.2 Emotion in AI-generated images

The exploration of emotion in AI-generated images, a field emerging at the intersection of affective computing and visual arts, has gained significant momentum. This interdisciplinary area investigates how AI can simulate and evoke human emotional responses through images, a development that reflects the growing sophistication of AI in understanding human emotions (Picard, 2003; Tao and Tan, 2005).

**Figure 1:**
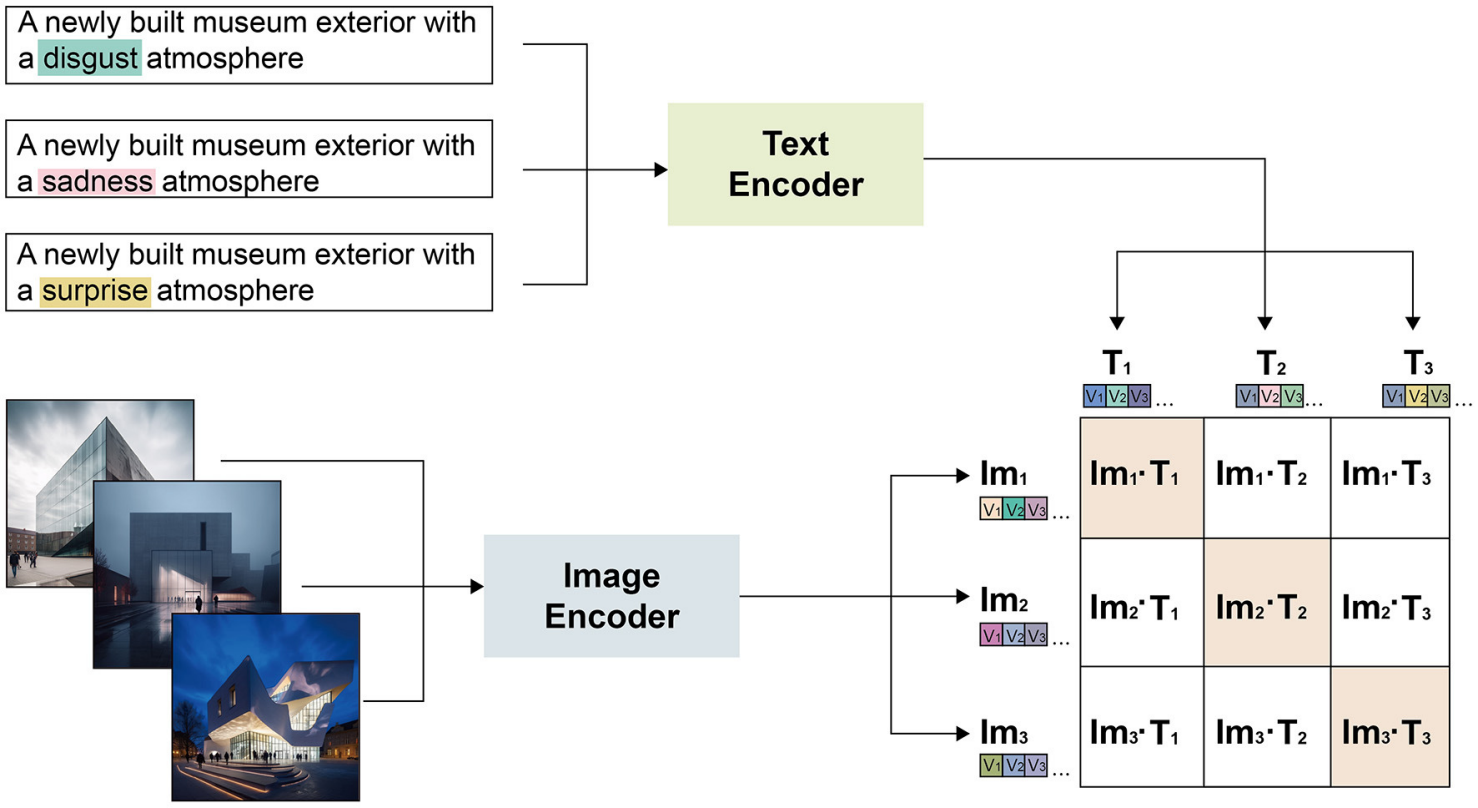
Technological advancements in emotionally resonant image generation: principles of techniques similar to CLIP for embedding emotional context into images.

Central to this domain is the capability of AI, particularly machine learning algorithms, to discern and replicate emotional cues in images. These algorithms, trained on extensive emotional datasets, enable AI to generate images that resonate with viewers, paralleling the emotional impact traditionally found in human-created art (Goodfellow et al., 2014; Sun et al., 2022; Gao et al., 2023). Projects like IBM's Watson and OpenAI's CLIP model illustrate AI's potential in creating emotionally engaging visual content (see Figure 1), utilizing advanced techniques to interpret and manipulate emotional content within imagery (Gatys et al., 2016; Radford et al., 2021).

Sentiment analysis, a critical component of affective computing, has been extended to the realm of AI-generated images. This involves algorithms interpreting the emotional tone of images, an approach particularly relevant in the analysis of architectural imagery. The emotional impact of design elements such as spatial composition, color schemes, and textural details can be explored through AI-generated visualizations, offering new insights into architectural design and its emotional resonance (Yildirim, 2022; Enjellina et al., 2023; Ploennigs and Berger, 2023).

However, generating emotional content in images through AI raises significant challenges and ethical considerations. Issues of authenticity in AI-generated emotional expressions and biases in AI-created imagery are major concerns (Zhang et al., 2023). The ethical implications of AI's potential to manipulate emotional responses, particularly in sensitive fields like architectural design, call for careful scrutiny (Chiarella et al., 2022; Futami et al., 2022). The interaction between AI-generated emotions and human responses in architectural imagery is an area of growing interest, with studies focusing on how these images influence human emotional and behavioral responses, and what this implies for the future of architectural experience (Viliunas and Grazuleviciute-Vileniske, 2022; Enjellina et al., 2023).

## 3 Research hypotheses

In exploring the application of AI in architectural design and its impact on emotional perception, this study aims to validate the following hypotheses, which are formulated based on prior research and theoretical frameworks. These hypotheses serve as the foundation for the study's design and methodology, guiding our investigation into the emotional role of AI in architectural visual representation and its impact across different audience groups.

**Hypothesis 1 (H1)**: AI-generated architectural images are capable of effectively eliciting specific emotional responses, demonstrating similar or superior emotive resonance compared to human-designed structures.

**Hypothesis 2 (H2)**: There is a significant difference in emotional perception of AI-generated architectural images between architecture students (Group A) and non-architecture students (Group B). This difference is attributed to the professional training of architecture students, making them more sensitive to the emotional details in the images.

**Hypothesis 3 (H3)**: AI-generated architectural images created within interior settings, such as homes, are more effective in expressing emotions compared to those generated in exterior settings, such as museums.

By systematically validating these hypotheses, we aim to contribute to the ongoing discourse on the integration of AI in architectural design, particularly in terms of enhancing emotional engagement and understanding among diverse groups.

## 4 Method

### 4.1 Software and tools selection

The choice of software and tools was crucial in our research aimed at examining how various AI technologies render emotional content in architectural imagery. We conducted an evaluation of five prominent image generation software: Stable Diffusion (Version 1.5 with LDMs Algorithms) (Pinaya et al., 2022), DeepFloyd IF (Stability, 2023), DALL E2 (Open, 2023), Midjourney (Version 5.1) (Midjourney, 2023), and Photoshop 2023 (Adobe, 2023). Each tool was tested using two prompts designed to generate images of a newly built museum exterior, with one prompt emphasizing a “happy atmosphere” and the other focusing on creating a “cheerful atmosphere that reflects happiness.” This approach allowed us to generate a collection of images for a comparative analysis of each software's ability to capture and convey the emotional essence of the prompts.

In our analysis, Midjourney distinguished itself by most accurately reflecting the intended emotional tones of the prompts. DeepFloyd IF demonstrated a somewhat limited correlation with the specified emotional content. Other tools, including Stable Diffusion, DALL E2, and Photoshop 2023, showed varying degrees of effectiveness in recognizing and rendering the emotional subtleties embedded in the prompts. Open-source platforms like Stable Diffusion and DeepFloyd IF offer extensive customization through plugins like ControlNet, providing detailed control over image generation aspects. The potential integration of technologies like Dreambooth and loRA with these platforms hints at future advancements in developing emotion-specific AI models. Conversely, DALL E2 and Photoshop 2023, while excelling in localized and extensive image modifications, did not align as effectively with our specific research focus on emotional expression in AI-generated architectural visuals.

We selected Midjourney as our primary tool, primarily due to its proficiency in generating images that resonated emotionally from text descriptions. This choice underscores our research intent to delve into AI's capability to evoke specific emotional responses through architectural imagery, a vital component in understanding the nuances of human-AI interaction within behavioral sciences.

### 4.2 Artificial intelligence in architectural rendering

Our research utilized the Midjourney AI software for generating images, focusing on two architectural settings: “home interior” and “museum exterior.” The choice of a “home interior” setting was driven by its universal relevance in daily life, providing a familiar context for eliciting and analyzing emotional responses. On the other hand, the “museum exterior” was selected for its cultural and public significance, offering a diverse spectrum of emotional engagement possibilities.

The crafting of prompts for AI image generation was a key element in our study. We aimed to explore how AI interprets and visualizes emotions within architectural contexts. To achieve this, we developed two types of prompts: one incorporating explicit emotional descriptors, such as “joy,” and another utilizing metaphorical language to convey emotions, like “creates a cheerful atmosphere that reflects happiness.” This dual approach allowed us to assess the effectiveness of both direct and metaphorical expressions in translating emotions into AI-generated architectural images.

**Figure 2:**
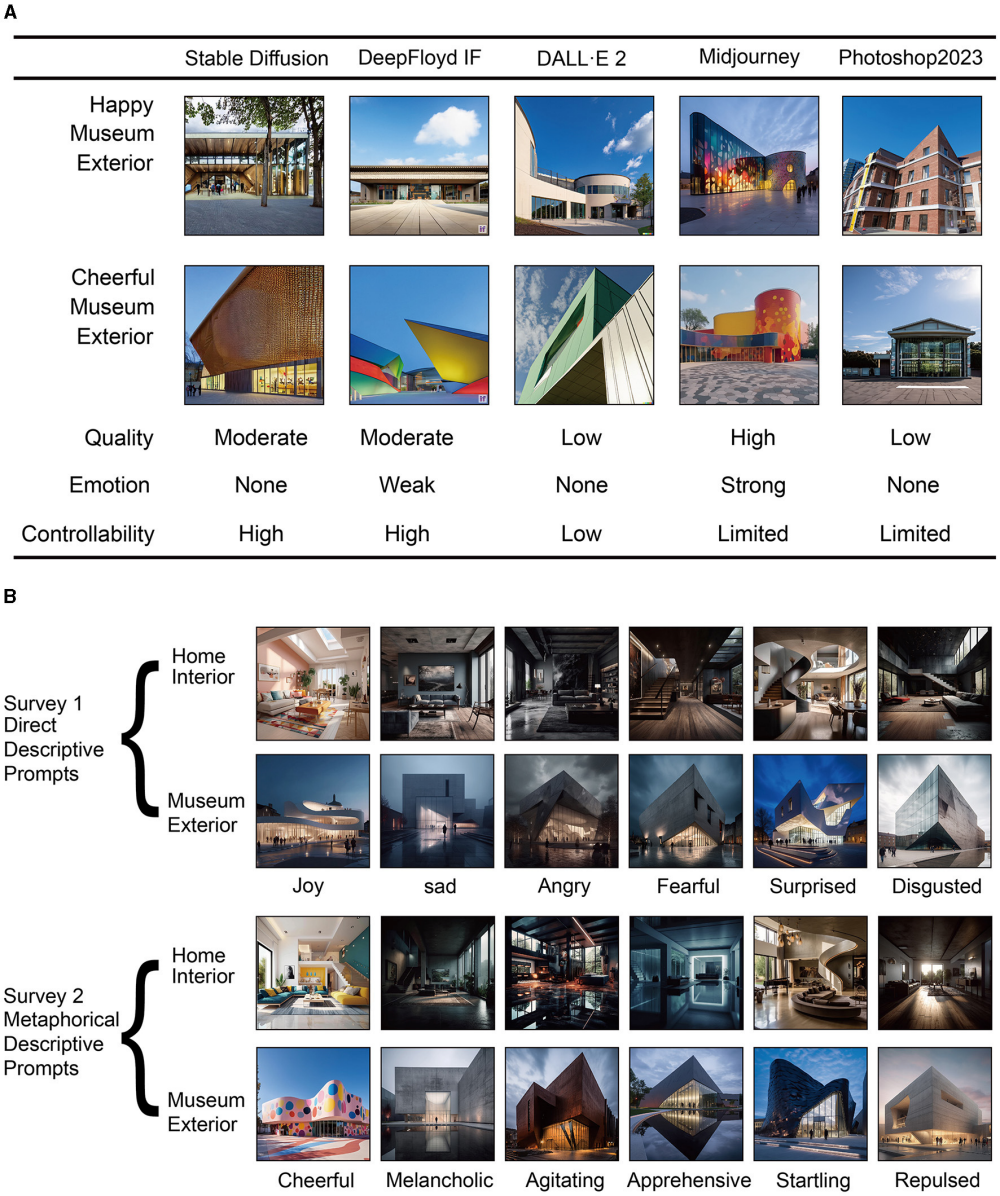
**(A)** Comparative analysis of image generation software, **(B)** architectural images generated through AI, illustrating the visual expression of the six basic emotions examined in the experiment.

Informed by Ekman's (2005) theory of basic emotions, our study encompassed six emotions: joy, sadness, anger, fear, surprise, and disgust. This range was integral to examining a wide array of emotional responses in architectural environments. For each emotion, we generated images for both the “home interior” and “museum exterior,” culminating in a diverse set of 24 architectural images (see Figure 2).

For example, to create imagery for a “museum exterior,” we employed prompts like “A newly built museum exterior with a happy atmosphere” to directly express the emotion and “a newly built museum exterior creates a cheerful atmosphere that reflects happiness” for a metaphorical representation. This method was consistently applied across different emotions and replicated for the “home interior” settings. The variation in emotional content of the prompts was crucial in our exploration of how AI-generated architectural renderings could mirror and potentially influence human emotions, a topic of great significance in behavioral sciences.

### 4.3 Survey design

The survey's design was a crucial element in our investigation into the emotional responses elicited by AI-generated architectural images. Our study aimed to discern the impact of these images on both architectural professionals and the general public, focusing on the psychological aspects of their reactions.

To optimize participant engagement and reduce fatigue, the survey was structured into two separate sections. Each section presented participants with a series of AI-generated architectural images. These images were aligned with specific emotions, conveyed either through direct or metaphorical language. Participants were instructed to rate their immediate emotional response to each image using a 0 to +10 scale. On this scale, 0 represented a very weak emotional response, while 10 denoted a highly intense reaction. This rating method was devised to encompass a broad spectrum of emotions, including happiness, sadness, anger, fear, surprise, and disgust. We encouraged participants to trust their gut reactions, underlining the subjective nature of the survey and affirming that there were no right or wrong answers. In designing our survey, we opted for a framework that participants could easily understand and engage with to assess the emotional responses elicited by AI-generated architectural imagery. Therefore, we employed the six basic emotions framework due to its clarity and ease of explanation to participants. Some more recent emotional theory frameworks, such as those proposed by Cowen et al. (2020) and Tang et al. (2023), offer a broader range of emotions and dimensions that, while providing detailed insights into emotional experiences, could potentially confuse participants in this study and significantly increase the workload involved in conducting the survey and analyzing the data. Therefore, we did not adopt these more complex emotional frameworks.

Furthermore, we decided against using a binary approach to emotional analysis, such as the positive and negative polarity, due to its limited capability in capturing the rich emotional engagement we aimed to explore with architectural imagery. In our previous experiments on emotional assessment, including the use of the Self-Assessment Manikin (SAM) questionnaire, participants indicated challenges in comprehension and the need for extensive explanation, impacting the efficiency and effectiveness of the survey (Zhang et al., 2022). Feedback from these preliminary trials revealed a preference among participants for the basic emotions model, which they found to be more intuitive and relatable. By choosing the six basic emotions, our study aimed to maintain a clear and consistent evaluative framework, effectively capturing the subtleties of people's emotional responses to AI-generated architectural imagery without the complexities and ambiguities associated with more elaborate emotional frameworks. Overall, the survey was meticulously designed to probe the intricate relationship between AI-generated images and human emotions, particularly in the context of architectural visualization. This methodology was central to our overarching goal of uncovering and understanding emotional reactions within architectural settings, thereby enriching the discourse in behavioral sciences.

### 4.4 Participants

In our study, the selection of participants was crucial for exploring the emotional and psychological responses to AI-generated architectural imagery. Inspired by Garip and Garip's (2012) findings, which indicate aesthetic differences between architecture and non-architecture students, we sought to investigate how such disparities might influence the perception and emotional response to AI-enhanced architectural visuals. To this end, we recruited 789 university students, aged 19–40, and divided them into two distinct groups: architecture majors (Group A, comprising 389 participants) and non-architecture majors (Group B, comprising 400 participants). This division was strategically chosen to assess the impact of educational and professional backgrounds on the engagement with AI-generated architectural imagery, grounding our participant selection in the premise that professional training and educational experiences significantly shape aesthetic judgment and emotional interactions with architectural design.

Group A, consisting of architecture students, was presumed to possess a deeper understanding and critical appreciation of architectural design. This expertise was expected to influence their emotional responses, with a potential focus on technical and aesthetic aspects of the AI-generated images.

In contrast, Group B included students from diverse non-architecture disciplines, representing a broader demographic akin to the general public. Their reactions were hypothesized to be more rooted in instinctual emotional responses, offering insights into how AI-generated architectural visuals are perceived by those outside the architectural field.

In adherence to the Sex and Gender Equity in Research (SAGER) guidelines, our study consciously did not collect gender-specific data, aiming to eliminate potential gender bias. This decision was made to ensure that our findings were focused solely on emotional and perceptual responses, irrespective of gender (Heidari et al., 2016).

The comparative analysis between these two groups was designed to provide a holistic understanding of how different educational backgrounds affect the perception and emotional engagement with AI-generated architectural imagery. Insights gained from this study are expected to contribute significantly to the fields of behavioral sciences and architectural design, particularly in understanding how AI-generated visuals are received and interpreted by diverse audiences.

### 4.5 Analysis strategy

The strategy for analyzing our data revolves around three core pillars, each designed to thoroughly investigate the role of AI in creating emotionally resonant architectural imagery. This tripartite approach allows us to delve into both the emotive capacity of AI-generated images and the perceptual differences in their reception among varied audiences.

#### 4.5.1 Assessment of emotive expressivity in AI-generated images

The first aspect of our analysis is dedicated to evaluating the emotional expressiveness of AI-generated architectural images. This involves examining how closely the emotions conveyed in the AI-generated prompts align with the emotions perceived by participants. By assessing this alignment, we aim to understand the effectiveness of AI in accurately interpreting and rendering the intended emotional content within architectural visualizations. This analysis is crucial in uncovering the psychological impact these AI-generated images have on viewers.

#### 4.5.2 Effectiveness of descriptive methods in emotional conveyance

Our second pillar concentrates on comparing the effectiveness of two descriptive approaches “direct descriptive words vs. metaphorical language” in AI-generated images. The goal here is to determine which method more effectively communicates the intended emotional context within the imagery. This comparison is vital for understanding the influence of language in shaping emotional perception in AI-generated architectural visuals.

#### 4.5.3 Differential interpretation between professional and lay audiences

The third pillar of our analysis distinguishes the perceptual differences between architectural professionals and the general public in response to AI-generated architectural images. This comparison seeks to gauge the utility of AI imagery as a tool for professional use in architecture, as well as its role in facilitating intricate and nuanced architectural representations. Analyzing the variances in emotional and perceptual responses between these groups offers insights into how AI-generated imagery is interpreted across diverse audiences.

In order to ensure a consistent rating scale across all images, we normalized the original average scores for each emotion. The normalized score rate *P*_*ij*_ for emotion *j* on image *i* is calculated as follows(refer to Equation 1):


(1) $$\begin{array}{l} P_{ij}=\frac{E_{ij}}{S_{i}\times n} \end{array}$$

where, *E*_*ij*_ is the original average score for emotion *j* on image *i*; *S*_*i*_ is the sum of the average scores for all emotions on image *i*; *n* is the number of images, which is six in our study.

This normalization process ensures that the sum of the scores for all emotions on each image equals 1/6, allowing for a fair comparison of the relative prominence of each emotion across different images. The normalized score rate *P*_*ij*_ reflects the proportion of the average score for emotion *j* relative to the average scores for all emotions on the given image.

In the analysis of data across different groups within our study, we adapted our statistical approach based on the specific characteristics of the dataset. For datasets exhibiting a normal distribution, the analysis was conducted using *t*-tests to compare means, alongside the calculation of Cohen's *d* to provide a measure of effect size, following the guidelines set by Schmidt and Bohannon (1988). In instances where the dataset deviated from normal distribution, the Wilcoxon signed-rank test was employed as a non-parametric alternative, with Cliff's (1993) delta utilized to assess the magnitude of the observed effects.

To facilitate our data analysis process, we leveraged a suite of Python libraries tailored for statistical computing and visualization. This included the use of NumPy for handling array-based numerical computations, Pandas for its powerful data structure and analysis tools, SciPy for conducting both parametric and non-parametric statistical tests, and plotly for creating visual representations of our findings. The integration of these tools not only bolstered the thoroughness of our statistical examination but also enhanced the clarity and interpretability of the results presented.

## 5 Result

### 5.1 Emotional expression across all groups

**Figure 3:**
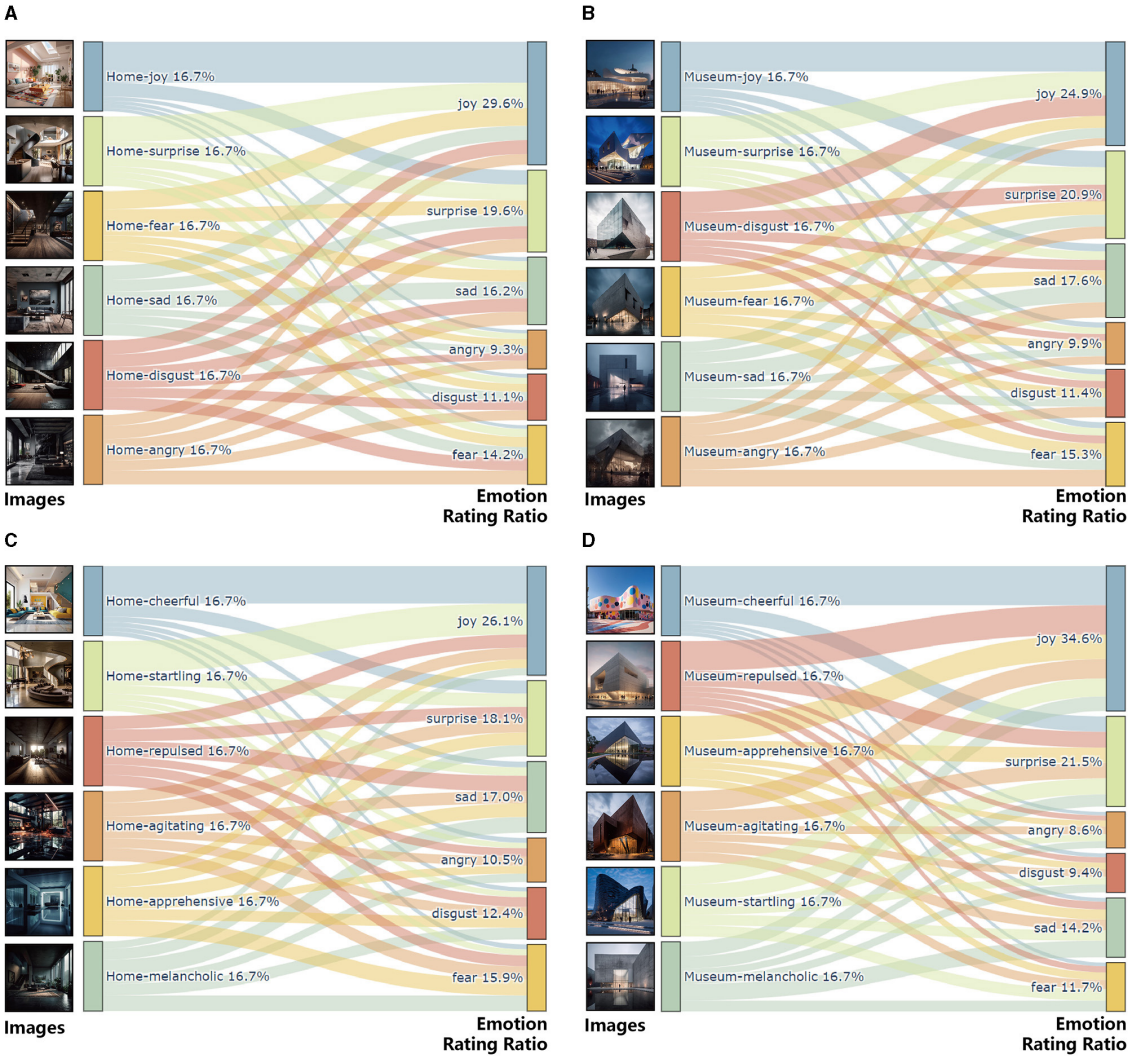
**(A)** Sankey diagram illustrating emotion ratings distribution for home images generated by direct prompts, **(B)** Sankey diagram illustrating emotion ratings distribution for museum images generated by direct prompts, **(C)** Sankey diagram illustrating emotion ratings distribution for home images generated by metaphoric prompts, **(D)** Sankey diagram illustrating emotion ratings distribution for museum images generated by metaphoric prompts.

Our comprehensive analysis of AI-generated architectural images across all participant groups revealed notable trends in emotional expression, which is illustrated in Figure 3.

#### 5.1.1 Direct prompt (home and museum settings)

In the direct prompt category for home settings, joy emerged as the most prevalently expressed emotion at 65.87%. The least effectively conveyed emotion was anger, registering only 11.02%. Other emotions like sadness, fear, surprise, and disgust ranged between 16.53 and 22.92%. In the museum settings under direct prompts, joy still led at 42.63%, but with a notable reduction in effectiveness compared to home settings. Disgust recorded the lowest effectiveness at 10.60%.

#### 5.1.2 Metaphoric prompt (home and museum settings)

With metaphoric prompts, joy remained the dominant emotion in home settings, scoring 57.19%. The lowest effectiveness was again seen in anger at 11.98%. For museum settings, joy's effectiveness slightly decreased to 56.05%, with disgust being the least effective at 7.72%.

#### 5.1.3 Patterns in highest-rated emotions

Our analysis of the highest-rated emotion for each image revealed some intriguing patterns:

- In home settings with direct prompts, joy dominated other emotions, even when the prompts were intended to evoke different emotions like sadness or fear.
- In museum settings, the results were more mixed, with joy still prevailing but to a lesser extent, indicating potential ambiguities in emotional expression.

#### 5.1.4 Positive and negative emotional performance

When categorizing emotions as positive and negative, we observed:

- In home settings with direct prompts, positive emotions like joy, surprise, and disgust outperformed negative emotions like sadness and fear.
- In museum settings, the performance gap between positive and negative emotions was narrower, with anger and disgust showing reduced effectiveness.

#### 5.1.5 Indoor vs. outdoor image analysis

The study also indicated that indoor images generally conveyed emotions more effectively than outdoor images. This trend was consistent across both direct and metaphorical prompts, suggesting that the spatial context significantly influences emotional perception in AI-generated imagery.

In summary, our results indicate a trend where AI-generated images are more effective in conveying positive emotions, particularly joy, across different settings and prompt types. The effectiveness of emotional expression also appears to be influenced by the architectural context, with indoor images demonstrating a higher capacity for emotional conveyance. These findings offer significant insights into the capabilities and limitations of AI in architectural visualization, particularly in its ability to resonate emotionally with viewers from diverse backgrounds.

### 5.2 Emotional expression across Group A and Group B

**Figure 4:**
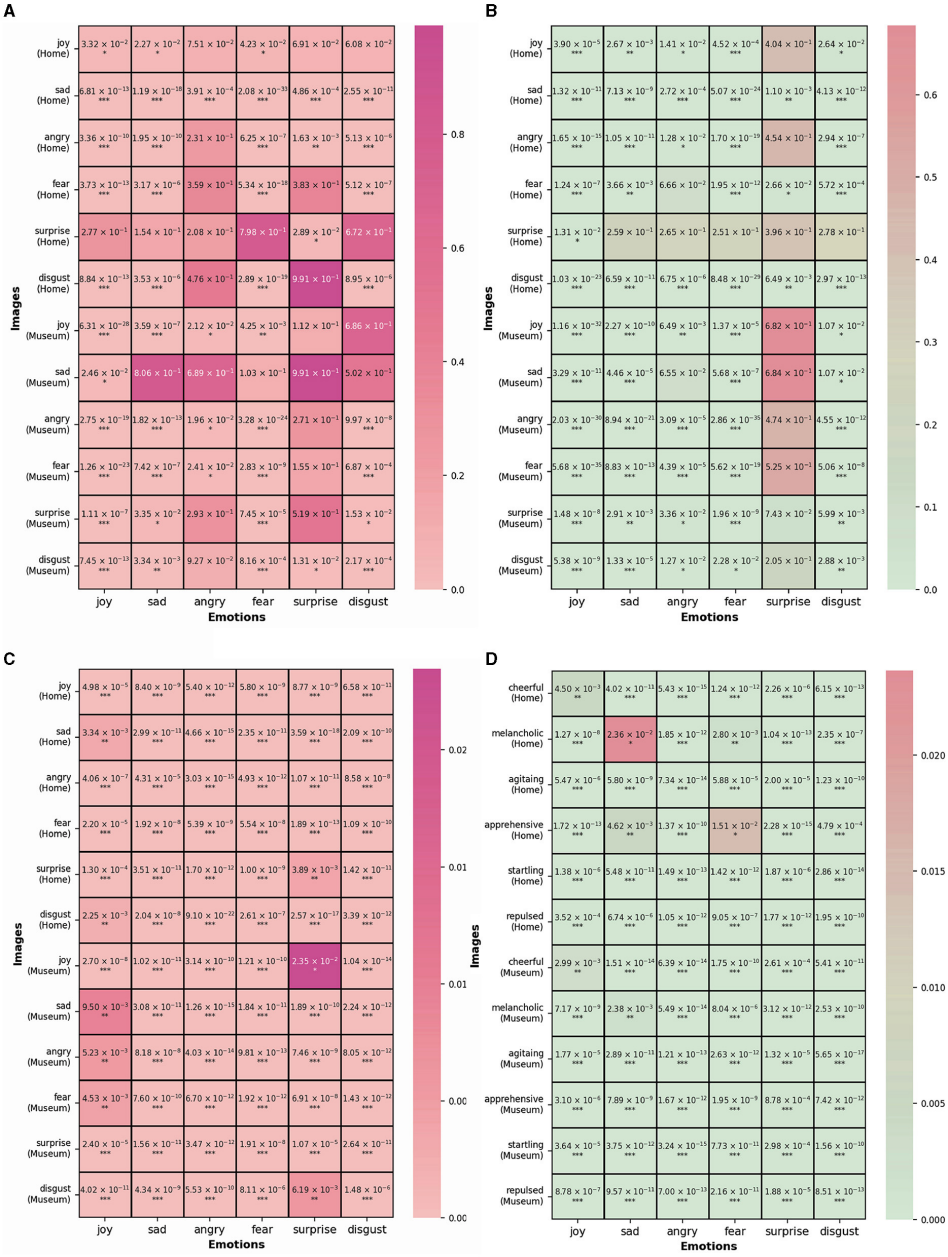
**(A)** Comparative analysis of emotion rating for Group A—heatmap of direct prompt vs. metaphoric prompt, **(B)** comparative analysis of emotion rating for Group B—heatmap of direct prompt vs. metaphoric prompt, **(C)** comparative analysis of emotion rating between Group A and Group B using direct prompt, **(D)** comparative analysis of emotion rating between Group A and Group B using metaphoric prompt. ^*^P-value < 0.05; ^**^P-value < 0.01; ^***^P-value < 0.001.

Our study's analysis of AI-generated architectural images revealed distinct patterns in emotional perception between architecture students (Group A) and non-architecture students (Group B), as outlined in Figure 4.

#### 5.2.1 Comparison between direct and metaphoric prompts

Within Group A, we observed minimal differences in the perception of surprise and sadness between direct and metaphorical prompts. This suggests a uniformity in emotional interpretation regardless of the prompt type for these emotions. Group B, however, showed a more pronounced difference in the perception of sadness across the prompt types, indicating varied emotional interpretations based on educational background.

#### 5.2.2 Direct prompt analysis

Comparing the emotional perception under direct prompts between the two groups, we noted significant differences across all emotions. The variance was particularly notable for joy and surprise (*P*-value of 0.0235), suggesting these emotions are universally perceived but with subtle differences influenced by the viewer's background. Metaphorical prompts also demonstrated differences in emotional scores between the groups, with the least variance in fear and sadness (*P*-value of 0.0236).

#### 5.2.3 Individual emotional expression

In Group A's direct prompt for home settings, joy was the most prominently expressed emotion at 66.01% Group B showed a similar trend, with joy being the most effectively expressed emotion. However, the overall performance of emotional expression in Group B was slightly lower than in Group A.

#### 5.2.4 Analysis of highest-rated emotion

In Group A's direct prompt (home), joy dominated the ratings even for images intended to convey other emotions like sadness or fear. The museum settings in Group A showed a mixed pattern, with joy still leading but with less dominance compared to home settings.

#### 5.2.5 Positive and negative emotional performance

The binary analysis revealed that positive emotions, particularly joy, were better conveyed in AI-generated images than negative emotions across both groups. The effectiveness varied depending on the setting, with indoor (Home) images generally showing better emotional rendering ability than outdoor (Museum) images. Overall, our results indicate significant differences in the emotional perception of AI-generated architectural images between architecture and non-architecture students. These findings provide valuable insights into how educational background influences emotional interpretation of AI-generated images, with implications for the utilization of AI in architectural design and its perception by different audience segments.

## 6 Discussion

This study provides an in-depth comparison of the emotional perceptions of two distinct groups toward AI-generated architectural images, unveiling several key findings and their broader implications.

### 6.1 The technical challenges of emotional expression

Our research delves into the significant challenges AI faces in encoding complex emotions into visual forms. While “joy” has been consistently and effectively depicted, our findings show that other emotions such as anger, sadness, and fear are less accurately portrayed. This discrepancy underscores the inherent difficulty of translating the nuanced spectrum of human emotions into AI-generated imagery. It highlights the imperative need for the development of more advanced AI models that can more finely understand and reflect the complexity of human emotions. For instance, the exploration of deep learning and neural networks presents a promising avenue to enhance AI's capability in emotion understanding and expression. These findings suggest a divergence from our initial hypotheses, indicating that while AI shows potential in emotional representation, its current abilities to capture and convey the full range of human emotions are limited.

### 6.2 The impact of educational background on emotional interpretation

Our research revealed significant differences in emotional perception between architecture students (Group A) and non-architecture students (Group B), aligning with our hypothesis. These differences can likely be attributed to the specialized training of architecture students, who are educated to understand the interplay between spatial design and emotional evocation. This finding highlights how educational and professional training shapes individuals' emotional interpretation of architectural spaces. It underscores the importance of interdisciplinary collaboration, integrating AI technology and emotional understanding in the educational process to provide designers and architects with a more comprehensive training. This synergy between AI and architectural education not only validates our initial hypothesis but also opens up new avenues for enriching the emotional depth of architectural design through AI.

### 6.3 The environmental impact on emotional rendering

Our study emphasizes the critical role of architectural imagery in eliciting emotional responses, with a notable finding that AI-generated images of indoor settings, such as homes, are more effective in emotional rendering than those of outdoor settings like museums. This distinction between indoor and outdoor environments in terms of emotional expression aligns with our hypothesis and is crucial for understanding the application of AI in architectural design. Emotional rendering, alongside aesthetic and functional considerations, plays a vital role in architectural imagery.

Expanding upon the differences in emotional expression between indoor and outdoor environments, our analysis delves into how specific features of these settings influence the conveyance and perception of emotions. The findings suggest that future research should employ a broader array of environmental samples to validate and further explore these insights. Designers can leverage this knowledge to optimize spatial design, enhancing emotional resonance within architectural spaces.

While these observations are consistent with our initial hypotheses, it is important to acknowledge the limitations of our experimental setup, particularly the range of environments we were able to include. A more extensive exploration of different settings is necessary to deepen our understanding of how environmental factors impact emotional responses. This future research direction could offer more nuanced insights into the complex interplay between AI-generated architectural imagery and human emotion.

### 6.4 Limitations and future directions

Despite the valuable insights provided, our study faces limitations due to the rapid evolution of AI image generation software and the selected environmental settings, which may not fully represent the diverse architectural contexts. The reliance on a limited number of prompts to explore emotional conveyance and the lack of detailed analysis on the influence of architectural training underscore areas for future investigation.

Future studies should expand the variety of prompts and environments to capture a broader spectrum of emotional responses. A more detailed examination of the impact of architectural education on emotional perception could offer deeper insights, considering factors such as the duration and specificity of training experiences.

Moreover, the binary approach to emotional analysis in our study simplifies the complex nature of human emotions. Future research should employ more nuanced methods to analyze the multidimensional aspects of emotional responses, possibly incorporating multisensory elements beyond the visual to enrich the understanding of architectural imagery's emotional impact.

By addressing these limitations, subsequent research can enhance our comprehension of AI's role in architectural design, potentially leading to the development of practices that resonate more profoundly on an emotional level with diverse audiences.

## 7 Conclusion

This study sheds light on the nuanced capabilities and limitations of AI in evoking emotions within architectural imagery, revealing AI's proficiency in depicting joy and its superior emotional rendering in indoor environments. Particularly, architecture students displayed enhanced sensitivity to AI-generated images, likely due to their specialized training. These findings underscore AI's potential in bridging technological innovation with human emotional experiences in architectural design, suggesting a future where AI not only enhances aesthetic appeal but also fosters emotionally resonant spaces. This research marks a significant step toward understanding AI's role in architecture, emphasizing the importance of integrating emotional intelligence in design practices to create spaces that resonate with human experiences.

## Data availability statement

The datasets presented in this study can be found in online repositories. The names of the repository/repositories and accession number(s) can be found at: 10.6084/m9.figshare.23896818.

## Ethics statement

The studies involving humans were approved by Ethics Committee of the Universitat Politècnica de Catalunya. The studies were conducted in accordance with the local legislation and institutional requirements. The participants provided their written informed consent to participate in this study. No animal studies are presented in this manuscript.

## Author contributions

ZZ: Writing – original draft, Visualization, Supervision, Software, Methodology, Formal analysis, Data curation, Conceptualization. JF: Writing – review & editing, Supervision, Conceptualization. LG: Writing – review & editing, Methodology, Data curation, Conceptualization.
